# Supplementary material for: A novel baculovirus-derived promoter with high activity in the baculovirus expression system
Source: PeerJ. 2016 Jun 28;4:e2183. doi: 10.7717/peerj.2183 (PMC4928464; doi:10.7717/peerj.2183)
Supplement: Table S1 [file peerj-04-2183-s001.docx]

| Primer name | Sequence (5’-3’) |
| --- | --- |
| ***Cloning*** |  |
| pSeL^F^ | ggatccgtatacatcatgtgtttgaacggcgactg |
| pSeL^R^ | gactagtatattgtagcaaaaatttaatttttgccaaaag |
| pSeS^F^ | ggatccgtatacatcatgtgtttgaacggcgactg |
| pSeS^R^ | gactagtgccaaaagacttaataaatcatcagc |
| pSeL-140^F^ | tagtataccgaatgtacaatattgttg |
| pSeL-140^R^ | ctgggtgtagcgtcgtaagc |
| pSeL-120^F^ | tagtatacgttcaataatcaataaacctctc |
| pSeL-120^R^ | ctgggtgtagcgtcgtaagc |
| polh-pSeL^F^ | atctcgaggttcaataatcaataaacctctctattatcctttgtaaattttttatttactttgttgtccattgctgatcat |
| polh-pSeL^R^ | ctgggtgtagcgtcgtaagc |
| ***qPCR*** |  |
| qF-AcMNPV-pol | gggtcaggctcctctttgc |
| qR-AcMNPV-pol | ttacgcagccatcacaaacac |

Table S1. Sequence of the primers employed in the study
